# An improved strabismus screening method with combination of meta-learning and image processing under data scarcity

**DOI:** 10.1371/journal.pone.0269365

**Published:** 2022-08-05

**Authors:** Xilang Huang, Sang Joon Lee, Chang Zoo Kim, Seon Han Choi

**Affiliations:** 1 Department of Artificial Intelligent Convergence, Pukyong National University, Busan, Korea; 2 Department of Ophthalmology, Kosin University College of Medicine, Busan, Korea; 3 Korea Innovative Smart Healthcare Research Center, Kosin University Gospel Hospital, Busan, Korea; 4 Department of Electronic and Electrical Engineering, Ewha Womans University, Seoul, Korea; Vellore Institute of Technology: VIT University, INDIA

## Abstract

**Purpose:**

Considering the scarcity of normal and strabismic images, this study proposed a method that combines a meta-learning approach with image processing methods to improve the classification accuracy when meta-learning alone is used for screening strabismus.

**Methods:**

The meta-learning approach was first pre-trained on a public dataset to obtain a well-generalized embedding network to extract distinctive features of images. On the other hand, the image processing methods were used to extract the position features of eye regions (e.g., iris position, corneal light reflex) as supplementary features to the distinctive features. Afterward, principal component analysis was applied to reduce the dimensionality of distinctive features for integration with low-dimensional supplementary features. The integrated features were then used to train a support vector machine classifier for performing strabismus screening. Sixty images (30 normal and 30 strabismus) were used to verify the effectiveness of the proposed method, and its classification performance was assessed by computing the accuracy, specificity, and sensitivity through 5,000 experiments.

**Results:**

The proposed method achieved a classification accuracy of 0.805 with a sensitivity (correct classification of strabismus) of 0.768 and a specificity (correct classification of normal) of 0.842, whereas the classification accuracy of using meta-learning alone was 0.709 with a sensitivity of 0.740 and a specificity of 0.678.

**Conclusion:**

The proposed strabismus screening method achieved promising classification accuracy and gained significant accuracy improvement over using meta-learning alone under data scarcity.

## Introduction

Strabismus is a common ocular disease characterized by misalignment of the eyes when focusing on an object [1]. Strabismus commonly occurs among children due to their less developed visual maturity. Nevertheless, strabismus can also happen at any age and has a serious effect on patients’ lives. Teens and adults who have traumatic brain injuries or stroke can develop strabismus [2] and they are more likely to suffer mobility challenges, depression, and systemic disease [3]. Furthermore, strabismus is the leading cause of amblyopia, which leads to irreversible permanent vision loss [4, 5]. For the above reasons, strabismus screening has become extremely important and indispensable as it is the first step in preventing the deterioration of eyesight and facilitating early diagnosis.

So far, strabismus screening is mainly conducted manually by ophthalmologists through many tests, such as the cover and uncover test, prism cover test, and the Hirschberg test. However, these tests largely rely on ophthalmologists’ experiences, which give rise to the screening results being subjective. To mitigate this issue, several works have suggested using image processing methods to conduct strabismus screening to help determine strabismus automatically. Almeida et al. [6, 7] adopted image processing methods and geostatistical functions to screen strabismus from face images that were obtained by shining a light on the cornea. The geostatistical functions were first used to identify and elicit the eye regions, and the image processing methods were later used to locate the limbus and corneal light reflex (CLR) based on the location of eye regions. The detection of strabismus was finally assessed by measuring the vertical and horizontal distance of the center of the reflection to the center of the limbus. Huang et al. [8] deployed a face detection model and facial landmark detector to first extract the eye regions, and subsequently applied automatic binarization methods to locate the pupil center to measure the position similarity of eyes for strabismus screening. Valente et al. [9] applied image processing methods such as Circular Hough Transform and Haar cascade classifier to videos featuring a cover test to identify the position of pupil and limbus, which were subsequently used in an eye-tracking procedure to measure eye movements for screening strabismus.

More recently, with the development of deep learning methods, several works have been proposed to leverage the learning ability of convolutional neural networks (CNNs) to perform strabismus screening automatically. Lu et al. [10] suggested using a two-stage network structure to complete strabismus screening, in which an eye region segmentation network was first used to extract the eye region images, and then a classification network was employed to perform the classification on the extracted images. Zheng et al. [11] utilized a similar network structure as [10], but they focused on using the primary gaze photographs to train the networks. Despite the excellent classification accuracies these works have achieved, they both require a large number (at least thousands) of target images (i.e., normal and strabismic images). In practice, collecting such a large amount of data is not a trivial task, and carefully annotating the data undoubtedly increases the labor burden of experts.

To circumvent the problem of collecting a large amount of data to train CNNs, meta-learning has emerged. Meta-learning, also known as “learning to learn”, aims to imbibe the model with the capability to adapt rapidly to new tasks with very few training images [12, 13]. Specifically, the underlying core idea of meta-learning is to train a CNN-based feature embedding network through an episodic training mechanism that imitates the low-data scenarios, so that it can generalize well to the target task given only a few training samples. Meta-learning approaches have been successfully applied to many fields such as skin disease identification [14], femur fracture classification [15], and hand-foot-mouth disease prediction [16]. Interestingly, meta-learning is usually used to compare with transfer learning. Between them, meta-learning focuses more attention on using episodic training with a few data (i.e., metadata) to discover a learning algorithm that can yield good generalization, whereas transfer learning requires thousands or more training samples from downstream tasks to learn new representations.

In terms of feature extraction, despite CNNs in meta-learning methods can extract distinctive features of eye regions, they lack the ability to use the specific features (e.g., iris position, pupillary light reflex) that can be inferred from the images to help determine strabismus. When the number of the training images is limited (e.g., less than five), the absence of fully utilizing the inference information of eye regions further hampers meta-learning to achieve good classification performance for screening strabismus. Thus, in this study, to improve the utilization of features from images under data scarcity, we propose a method that combines a meta-learning approach with image processing methods for strabismus screening. The meta-learning approach was first pre-trained on a public dataset to obtain a well-generalized embedding network for feature extraction of eye regions. Meanwhile, the image processing methods were used as a position feature extractor to extract the eye region information neglected by the embedding network, so as to make full use of the feature information that can help strabismus screening given only a few training images.

## Materials and methods

### Ethics statement

This study adhered to the tenets of the Declaration of Helsinki, and the Institutional Review Board of the Gospel Hospital of Kosin University (KUGH-IRB 2020–03-034) approved the images used in this study. Informed written consent was obtained from all of the participants after giving a clear and elaborate explanation by the ophthalmologist including the purpose of the study and the image usage. For minor participants, written consent was obtained through their parents or guardians. In addition, participants who have strabismus were informed that their participation in the study would not affect the provision of ophthalmic care.

### Data set

In this study, 30 strabismic and 30 normal subjects were invited to participate in the experiment. The subjects include children (normal: 3; strabismus: 20) and adults (normal: 27; strabismus: 10). They underwent screening tests conducted by a professional ophthalmologist and the screening results were used as the ground truth to evaluate the classification results. The subjects followed the instructions of the ophthalmologist to complete the data collection. Each subject was asked to look straight at the camera (EOS 750D, Canon Inc., Tokyo, Japan) to take a frontal face image (containing only the majority of the face) from 1 m. The camera was equipped with a pen torch to obtain CLR in the pupil. Among all the participants, only 17 strabismic subjects and 22 normal subjects consented to provide the frontal image with CLR, and the remaining subjects agreed to provide only the frontal images. Thus, our data set consists of 30 normal images (no CLR:8; with CLR: 22) and 30 strabismic images (no CLR:13; with CLR: 17).

### The proposed strabismus screening method

**Fig 1:**
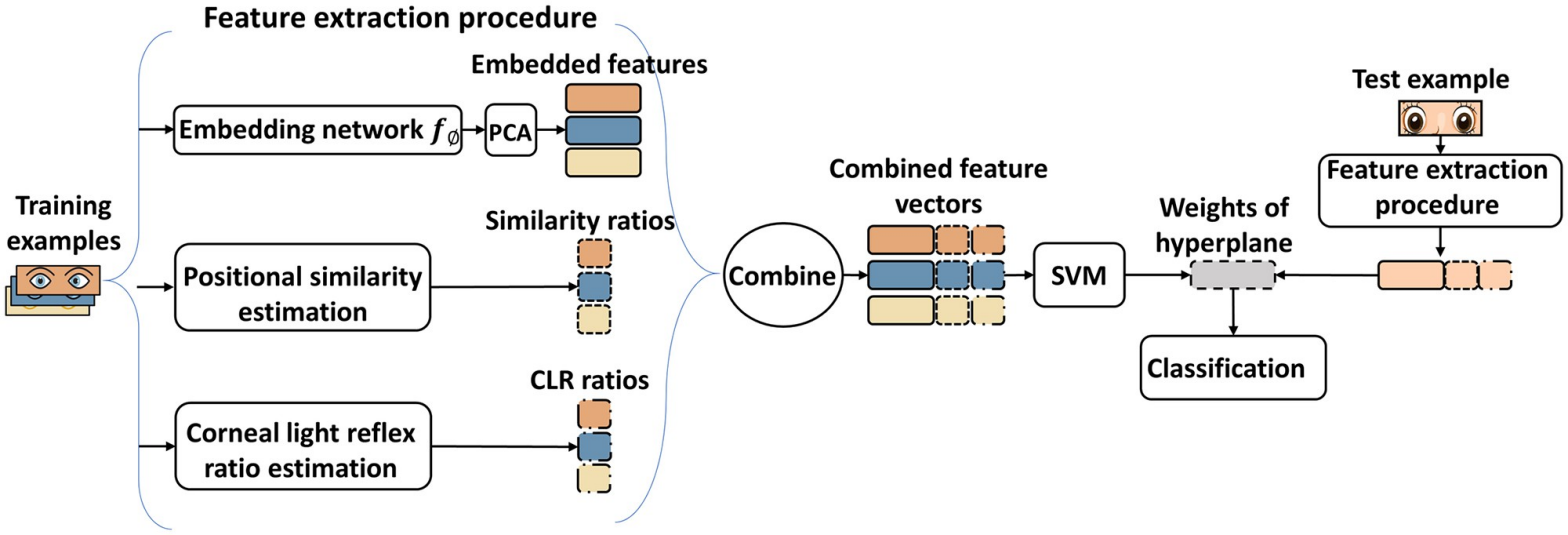
The proposed method for strabismus screening. The eye region training samples are sent to a pre-trained embedding network *f*_*ϕ*_ to extract significant features and subsequently are passed through principal component analysis to obtain low-dimensional embedded features. Simultaneously, position similarity estimation and corneal light reflex ratio estimation module are applied to the eye region samples to extract position information of eye regions. The information is combined with the embedded features to enrich the representational ability of features (to gain more information about the eye region) before training a support vector machine.

The proposed method combines a meta-learning approach called MetaOptNet [17] with image processing methods. Fig 1 shows the overall structure of the proposed method. Intuitively, the method was developed by inserting two image processing modules (i.e., position similarity estimation and CLR estimation) into the architecture of MetaOptNet to utilize the position information of eye region images fully. The training samples were the eye regions of normal and strabismic images, which were automatically extracted from the face images through the face detection model and the face mark detector. The details of eye region extraction will be introduced in the image processing section. The feature extraction procedure was composed of an embedding network *f*_*ϕ*_ followed by a principal component analysis (PCA) module, a position similarity estimation module, and a CLR ratio estimation module, in which the latter two modules were implemented by image processing methods. The embedding network obtained by the pre-trained MetaOptNet was used to extract embedded features of eye region images, whereas the image processing modules were used to extract the position features from eye regions as supplementary features to the embedded features. Among the supplementary features, the similarity ratio was a value that describes the position similarity between irises, whereas the CLR ratio was another value that measures the ocular alignment. Due to the dimensionality of embedded features obtained by the embedding network being much higher than the supplementary features, we applied PCA to embedded features to reduce their dimensionality before combining them with supplementary features, aiming to prevent the embedded features from dominating the supplementary features while retaining valuable information. Finally, a support vector machine (SVM) classifier learned a hyperplane based on the combined features to classify normal and strabismic images as accurately as possible.

### The architecture of MetaOptNet

**Fig 2:**
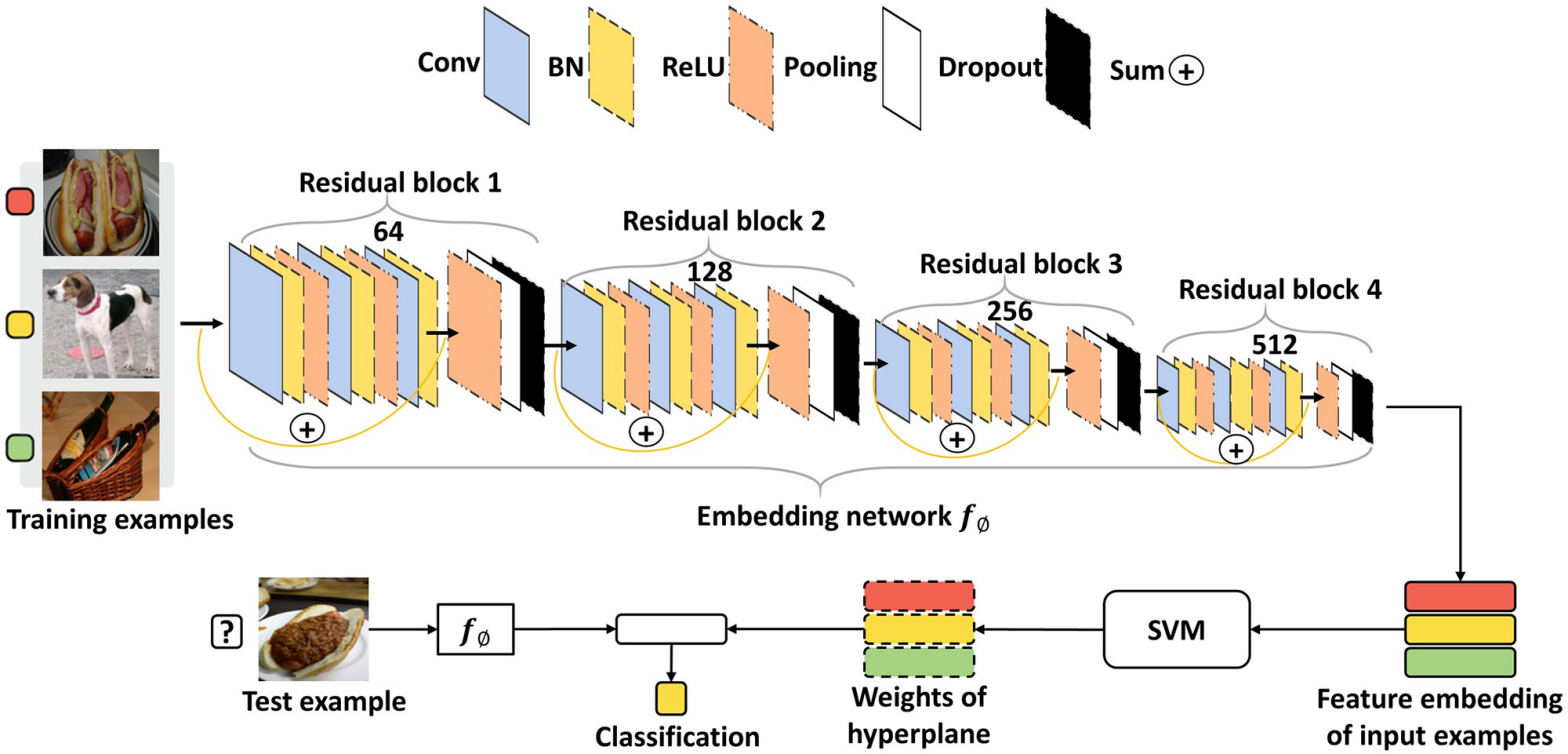
The detailed architecture of MetaOptNet. The goal of MetaOptNet is to adjust the parameters *ϕ* of the embedding network *f*_*ϕ*_ through various tasks so that it can generalize well to new tasks. The numbers (e.g., 64, 128, 256, and 512) represent the number of convolutional filters used in each residual block. The sum operation sums up the input of a residual block to the output of the third BatchNorm layer in the corresponding residual block. The images of training and test samples are obtained from the miniImageNet dataset, which is publicly available on https://drive.google.com/file/d/1fJAK5WZTjerW7EWHHQAR9pRJVNg1T1Y7/view.

In this study, we adopted the meta-learning MetaOptNet for the proposed method, and its detailed architecture is shown in Fig 2. Due to the limited number of strabismic and normal images, we first pre-trained MetaNetOpt using the miniImageNet dataset [18] in a few-shot manner to imitate the circumstance of data scarcity, which aims to obtain a well-generalized embedding network *f*_*ϕ*_. We followed the commonly-used split method [19] of the miniImageNet dataset, in which 100 classes are randomly split into 64, 16, and 20 classes as a meta-training set, meta-validation set, and meta-testing set, respectively. Each set plays a different role in meta-learning. The meta-training set is used to train the embedding network to learn good generalization parameters by minimizing the loss of misclassification, whereas the meta-validation set is used to select the best embedding network with the highest classification accuracy after each training iteration. The meta-testing set is used to evaluate the classification accuracy of novel classes after meta-training and meta-validation. However, because pre-training MetaOptNet using meta-training and meta-validation set was sufficient to fulfill the purpose of obtaining a well-generalized embedding network to extract significant embedded features in eye regions, we do not use the meta-testing set of miniImageNet in this study. To obtain the pre-trained MetaOptNet, we followed the episodic training mechanism to generate a support set (training samples) and a query set (test samples) in each training and validation iteration. The support set and the query set are usually composed of the same classes (usually 5 classes), each class in the support set has a few labeled samples while the query set contains unlabeled samples of each class. The samples in the support set do not intersect with the query set. Fig 2 shows the detailed architecture of MetaOptNet with images from the miniImageNet dataset. The support set in both the meta-training set and the meta-validation set was used to determine the hyperplane of SVM to classify samples from the query set. The query set in the meta-training set was used to construct the loss function of SVM classification for updating the network parameters, while in the meta-validation set, it was only used to evaluate the classification performance to select the best embedding network.

In MetaNetOpt, we used the ResNet-12 network [20] as the embedding network to learn to extract significant features from images. ResNet-12 consists of four residual blocks, each block includes three 3 × 3 convolution with *k* (e.g., 64, 128, 256, 512) filters, followed by batch normalization, Leaky ReLu(0.1). The final ReLu layer in each block is followed by a 2 × 2 max-pooling layer to extract the sharpest features and down-sample the features. DropBlock regularization [21] with a block size of 1 is applied to the last layer of the first two residual blocks, and the block with a size of 5 is applied to the last two residual blocks to improve the performance. ResNet-12 outputs an embedded feature with a dimension of 12,800 for each image, and SVM determines the optimal hyperplane from these features for classification.

**Fig 3:**
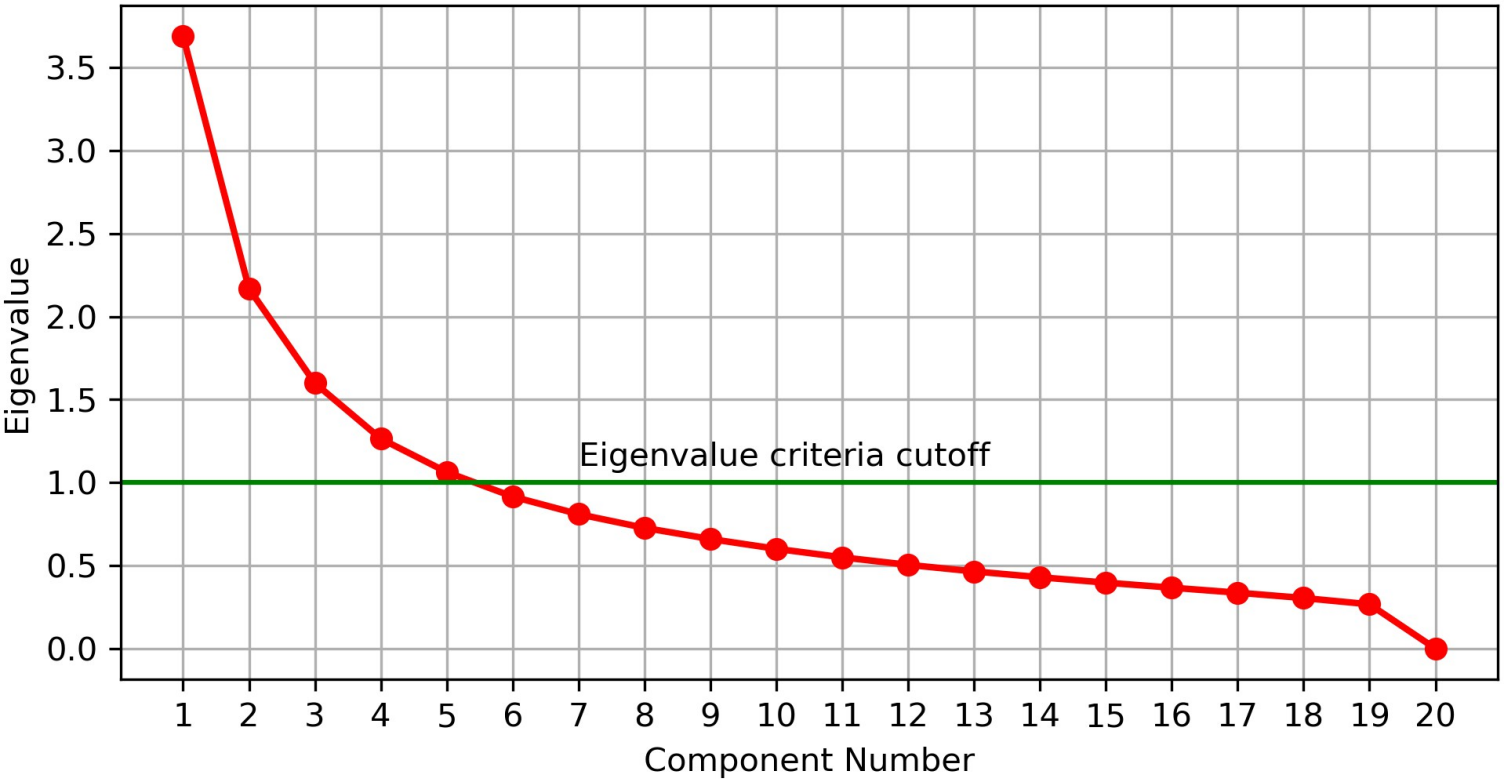
Scree plot for selecting the appropriate number of principal components for PCA.

As the dimensionality of embedded features is much higher than that of the supplementary features (i.e., one dimension), high-dimensional embedded features tend to dominate the semantics of their combination with supplementary features. Thus, we reduced the dimensionality of embedded features before combining them with supplementary features. Specifically, we determined the appropriate number of principal components for dimensionality reduction by setting the number of principal components of PCA to 20 in 10,000 embedded feature extraction experiments. From the experiments, we calculated the average of the eigenvalues of each principal component to obtain the scree plot (Fig 3), which is a graphical tool used to determine the number of principal components to be considered in PCA. As can be seen from Fig 3, only five principal components have eigenvalues greater than 1, which means that the principal component would explain more than one variable’s worth of the variability [22]. In other words, principal components with an eigenvalue greater than 1 contain more valuable information about the original embedded feature. In addition, the eigenvalue changes of the principal components with eigenvalues less than 1 have gradually stabilized relative to the first five principal components. Thus, the number of principal components used in the PCA module was determined as 5.

### Image processing on the eye region

**Fig 4:**
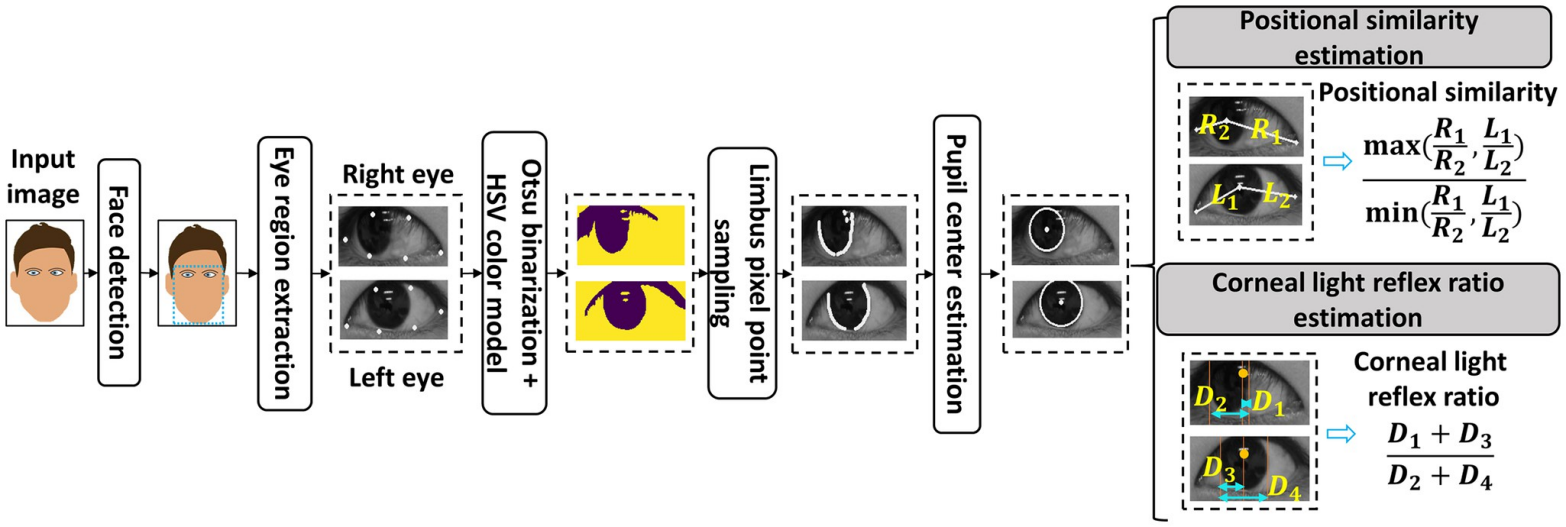
Image processing procedure on the eye region. *R*_1_, *R*_2_ and *L*_1_, *L*_2_ are the distance between estimated pupil centers and landmarks of the medial and lateral canthus on the right and left eye. *D*_1_ and *D*_3_ are the distances from the corneal reflex centers (orange dots) to the medial corneal limbus, *D*_2_ and *D*_4_ are the diameters of the iris.

After pre-training, MetaOptNet is capable of extracting the significant features from eye region images. However, the information on the eye region is still not fully utilized for strabismus screening. For example, the position information of irises and the CLR ratio could be used as supplementary features to describe the ocular deviation, which cannot be obtained from the embedding network. Therefore, we developed an image processing-based information extraction procedure for eye regions, which is shown in the flowchart in Fig 4. The procedure initially applied a face detection model and facial landmark detector provided by python-Dlib library [23] to identify and extract the eye region from a face image automatically. In detail, the face detection model returned the coordinates of a bounding box that enclosed the facial parts, which is shown in the resulting image after the face detection module. Based on the coordinates, the landmark detector located and extracted the eye region using six landmarks, which are shown in the resulted images after the eye region extraction block. Among these landmarks, the landmarks that lie on medial and lateral canthus were later used in calculating the position similarity. In the next stage, an automatic image thresholding method, Otsu’s binarization [24], and one of the widely used segmentation methods, the HSV color model [25], were separately implemented in the eye region to remove the background (e.g., skin color, sclera) while retaining most of the iris region. Otsu’s binarization automatically determines the optimal threshold value for converting an RGB image into a grayscale image by traversing all possible thresholds, whereas HSV uses the user-defined upper and lower bound color of Hue, Saturation, and Value to extract a region of interest. To adapt the HSV model color automatically to various input images, the lower and upper bound were set as [0, 0, 0] and [180, 255, v], where v is automatically determined by the average gray value of the eye region image. The resulted images from the two methods were formed into one image based on their common parts, which are shown in the output of the combination block of Otsu’s binarization and HSV color model in Fig 4. After acquiring the clear binary images of two eyes, we sampled the pixel points located at the limbus from each image in a bottom-to-top manner so that all of the pixel point samples were collected. Using the sampled pixel points, we applied the least square method (LSM) for circle fitting [26] to these points and obtained the pupil center for each eye, which can be seen in the resulted images after the pupil center estimation block in Fig 4.

### Position similarity estimation

With the pupil center, we separately estimated the position similarity of irises and the CLR ratio, which were used as supplementary features in addition to the embedded features. For position similarity estimation, the medial and lateral canthus landmarks obtained in the eye region extraction were used to compute their distance to the pupil center. We denote the distances between estimated pupil centers and the medial and lateral canthus of the right and left eyes as *R*_1_, *R*_2_ and *L*_1_, *L*_2_, respectively. These distances were further utilized to compute the ratios $\frac{R_{1}}{R_{2}}$, $\frac{L_{1}}{L_{2}}$ to provide position information of the iris in each eye. Then, the ratio with greater value (i.e., max($\frac{R_{1}}{R_{2}}$,$\frac{L_{1}}{L_{2}}$)) was divided by the smaller one (i.e., mim($\frac{R_{1}}{R_{2}}$,$\frac{L_{1}}{L_{2}}$)) considering the symmetry of both eyes. Thus, the position similarity of the iris can be defined as:

$$\begin{array}{c} S=\frac{max(\frac{R_{1}}{R_{2}},\frac{L_{1}}{L_{2}})}{mim(\frac{R_{1}}{R_{2}},\frac{L_{1}}{L_{2}})}. \end{array}$$ (1)

**Fig 5:**
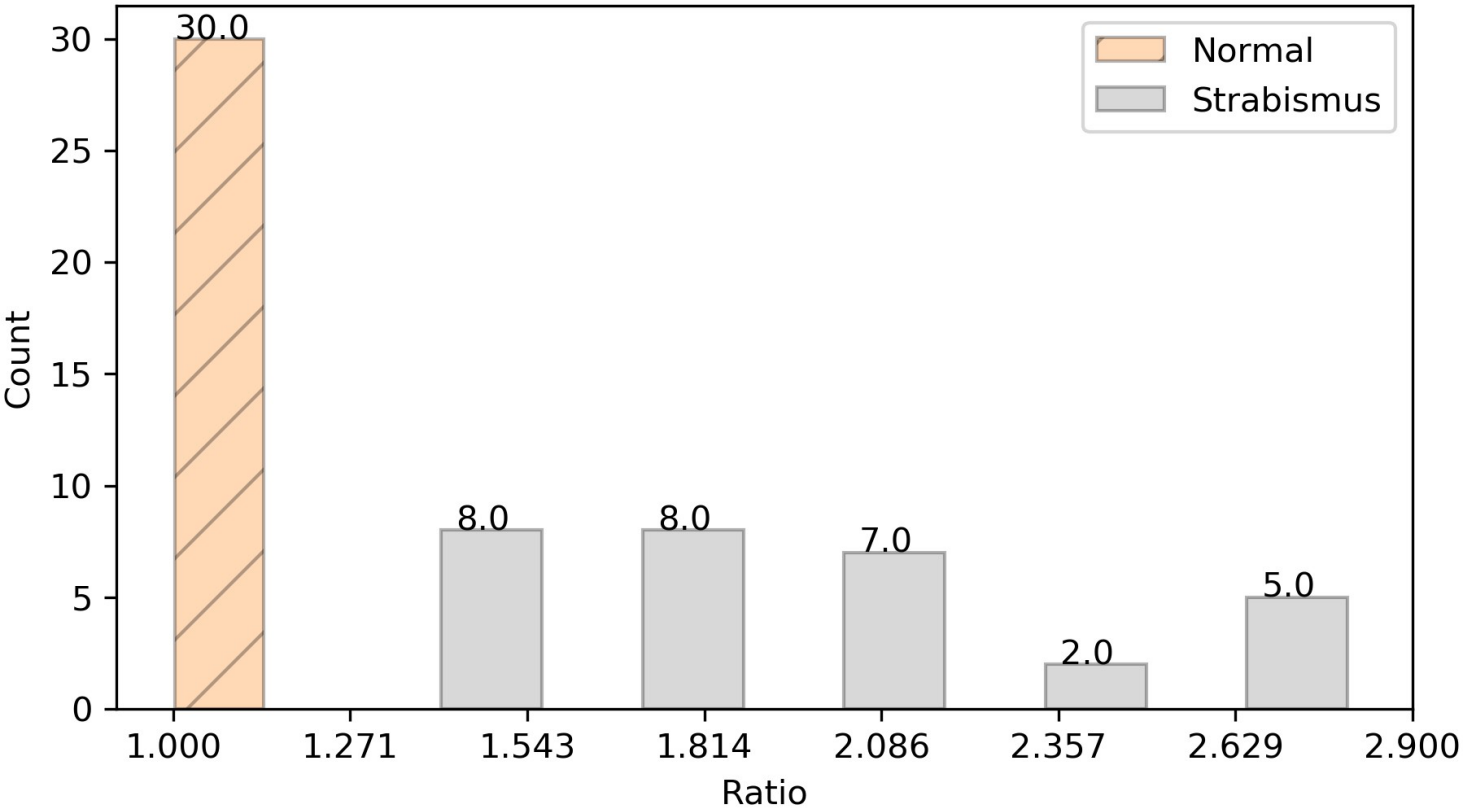
Distribution of position similarity ratio for normal and strabismic images.

If the resulted value is equal to or close to 1, the irises are in a similar and symmetrical position with each other, which means that the eyes are likely to be normal, otherwise the patient might have strabismus. The position similarity estimations of 60 images are shown in Fig 5.

It can be seen from Fig 5 that the position similarity ratios of normal images fall within a small range from 1.002 to 1.139, in which both boundary values are close to 1, indicating the effectiveness of position similarity estimation. On the other hand, owing to the existence of diverse types of strabismus in strabismic images (i.e., esotropia, exotropia, hypertropia, and hypotropia), the position similarity ratios of strabismic images are widely distributed in five ranges. Among them, eight images are in the range from 1.333 to 1.642, eight images are in the range from 1.642 to 1.951, seven images are in the range from 1.951 to 2.260, two images are in the range from 2.260 to 2.569, and five images are in the range from 2.569 to 2.877.

### Corneal light reflex ratio estimation

To estimate the CLR ratio, we adapted the method introduced in [27]. Notably, [27] used Adobe Photoshop software to manually process the eye images and measure the iris diameter and the distances between the light reflex to the corneal limbus. In contrast, we developed an image processing-based system to obtain the above measurements in an automatic manner, which can be seen in Fig 4. Concretely, we first located the reflection points on the iris region with Otsu’s binarization method and computed the average value of these points to obtain the center coordinate. Then, we used the iris radius obtained from the pupil center estimation module to add and subtract the pupil center coordinates to locate the corneal limbus coordinates. Finally, we adapted the formula $\frac{\left(D_{1}+D_{3}\right)}{\left(D_{2}+D_{4}\right)}$ to estimate the CLR ratio [27], in which *D*_1_ and *D*_3_ are the distances from the center CLR points to the corneal limbus, and *D*_2_ and *D*_4_ are the diameters of the irises. Among the 60 images, we observed some images cannot calculate the CLR ratio due to the absence of CLR points in the iris region. To deal with this issue, we utilized the normal and abnormal range of CLR ratios from [27], which is shown in Table 1. For training samples, the CLR ratio of a normal image with no CLR point was set to the medium value of the normal CLR ratio range (i.e., 0.468), and the CLR ratio of a strabismic image with no CLR point was set to 0. For test samples, because the labels are not known in advance, we used the median value between the maximum similarity ratio of normal images and the minimum similarity ratio of strabismic images in the training samples as the criterion to determine the CLR ratio. The CLR ratios of images whose similarity ratios *S* are smaller than or equal to the criterion were set to 0.468, otherwise, they were set to 0. Supplementary features were assembled with embedded features processed by PCA and standardization was applied to rescale the combined features to have a mean of 0 and standard deviation of 1 before training the SVM.

**Table 1:** Normal and abnormal range of CLR ratio [27].

Parameter	Measurement
Normal range	0.448–0.488
Abnormal range	<0.440, >0.497

### Experimental design

To obtain the pre-trained MetaOptNet, we randomly selected 5 classes and 10 samples of each class from the meta-training set of the miniImageNet dataset to form the support set in each training iteration. A query set was generated by randomly selecting 6 samples from the same classes as the support set. Thus, during the pre-training stage, the total training and test samples in each meta-training iteration were 50 and 30 images, respectively. When the total number of training iterations was exhausted, we applied the same settings to the meta-validation set to evaluate the current embedding network. During meta-validation, if the classification accuracy of the current embedded network is higher than the previous one, the current network is selected as the best one and the network parameters were stored. The drop rate of the DropBlock was set to 0.1 during the training procedure, which means that the features corresponding to the size of the block on the feature map have a 10% probability of being zero. The network used the stochastic gradient descent as the optimizer with a Nesterov momentum of 0.9 and weight decay of 0.0005. The size of the mini-batch was set to 2. The training epoch was set to 40, with each epoch consisting of 1,000 iterations. The learning rate was initially set to 1 and then changed to 0.6, 0.12, and 0.024 when the epoch was 10, 20, and 30, respectively.

After pre-training, we loaded the best parameters for the embedding network and evaluated the proposed method using 30 normal and 30 strabismic images collected from the participants. Specifically, in the feature extraction procedure of each experiment, we randomly selected 15 images from the normal and strabismic images as training samples to extract embedded features, similarity ratios, and CLR ratios to help train SVM for finding the classification hyperplane. The test samples consisted of the remaining images (i.e., 15 normal and 15 strabismic images) used to evaluate the classification accuracy. The performance of the proposed method was assessed by averaging the classification accuracy through 5,000 experiments. All face images were resized to a resolution of 1920 × 1280 to reduce the computational cost before estimating position similarity and CLR ratio. The eye region of each face image was automatically cropped by the face detection model and facial landmark detector and was resized to the resolution of 84 × 84 to fit the input of the embedding network. The proposed method was implemented based on Python (Python Software Foundation, version 3.7.9), PyTorch (Facebook, version 1.6.0), and OpenCV (Intel, version 3.4.2). The models were trained with an eight-core AMD Ryzen 7 2700 CPU and NVIDIA GeForce TRX 2080 Ti GPU.

## Results

The experiments were conducted strictly according to the guideline [28], and the experimental results were accurately reported in Table 2. The performance of MetaOptNet in strabismus screening was used as a baseline for comparison with the proposed method. To evaluate the performance of MetaOptNet and the proposed method, specificity was defined as the percentage of normal images that were correctly classified as normal, and sensitivity was defined as the percentage of strabismic images that were correctly classified as strabismus. MetaOptNet had achieved an average classification accuracy of 0.709 (95% confidence interval [CI] = 0.707–0.711) with a sensitivity of 0.740 (95% CI = 0.737–0.744) and a specificity of 0.678 (95% CI = 0.675–0.681). On the other hand, the proposed method had achieved a much higher classification accuracy of 0.805 (95% CI = 0.803–0.806) with a sensitivity of 0.768 (95% CI = 0.766–0.771) and a specificity of 0.842 (95% CI = 0.839–0.844).

**Table 2:** The classification performance of MetaOptNet and proposed method in strabismus screening.

Method	Accuracy (95% CI)	Sensitivity (95% CI)	Specificity (95% CI)
MetaOptNet	0.709 (0.707 to 0.711)	0.740 (0.737 to 0.744)	0.678 (0.675 to 0.681)
Proposed method	0.805 (0.803 to 0.806)	0.0.768 (0.766 to 0.771)	0.842 (0.839 to 0.844)

## Discussion

The traditional clinical strabismus screening methods such as the prism cover test and the Hirschberg test largely rely on the experiences of ophthalmologists, making the screening results subjective. To reduce the subjectivity of strabismus screening, many works [6–9] have suggested using image processing approaches to tackle the problem automatically. These works have demonstrated that the image processing methods can accurately extract desired features for strabismus screening based on the specific system design. Apart from the image processing-based methods, deep learning methods [10, 11] focuses on leveraging the learning ability of CNNs to learn the automatic location of eye regions and performing classification based on the eye regions. However, the performance of deep learning methods largely relies on using a large number of images to train CNNs and classifiers. In practice, the number of strabismic images is usually limited and needs to be carefully annotated by experts before training the networks, which greatly restricts the above methods to obtain effective CNNs and target classifiers for screening strabismus when the data are limited.

To overcome the data scarcity problem when using CNNs for strabismus screening, we presented a method that combines the meta-learning approach named MetaOptNet with image processing methods. Taking the benefits of the episodic training mechanism of meta-learning, MetaOptNet is capable of generalizing the network to strabismus screening tasks with a few normal and strabismic images. From Table 2, it can be seen that the sensitivity when using MetaOptNet alone is higher than the specificity. We suspect this phenomenon because SVM tended to draw the hyperplane in a high-dimensional space that focuses on the widely distributed embedded features from strabismic images than the features obtained from normal images. However, due to the nature of CNNs, MetaOptNet can only extract pixel information of images, which limits the efficiency of image information usage. To this end, the proposed method adopted image processing methods to extract the position information of eye regions as the supplementary features for increasing the discrimination between features of normal and strabismic images obtained from the embedding network. The effectiveness of the proposed approach was verified by improving the classification accuracy on both normal and strabismic images compared to using MetaOptNet alone. The underlying reasons for the experimental results of the proposed method can be summarized in two folds. First, the embedded features obtained from using MetaOptNet alone do not contain any information that can indicate the position of the eyes in the eye images, and tend to overlap with each other in the embedding space, making it difficult for SVM to determine an optimal hyperplane to classify normal and strabismic images. In contrast, the proposed method used the supplementary features to enrich the embedded features, thereby reducing the overlap between normal and strabismic features in the embedding space. Second, the supplementary features provide additional information for the embedded features, helping the SVM to simultaneously focus on the clustered features in normal images when drawing the classification hyperplane based on the widely distributed strabismus features, thereby achieving an overall improvement in classification accuracy.

Another aspect worth discussing is that the image processing methods in this work were adopted from our previous work [8]. However, we further developed a CLR estimation module based on the previous work to measure the reflection points of images to indicate the ocular alignment, which along with the position similarity obtained from the previous work are used as the supplementary features for the proposed method. These two supplementary features allow the proposed method to maximize the use of image features to aid in strabismus detection when data is insufficient.

### Limitations and strengths

The present study has some limitations. First, the image data only comes from a hospital in Busan; whether the result can be generalized to other regions remains to be verified. Second, images without CLR are the potential factors that affect classification performance. Therefore, more images with CLR need to be collected for further analysis. Third, position similarity estimates of normal images have a range from 1.002 to 1.139, in which the maximum value is slightly larger than 1. This may be caused by the imprecise localization of the medial and lateral canthus due to the factors such as illumination, eyelashes, and inapparent facial contours.

Despite the above-mentioned limitations of this study, the promising results illustrated the potential of the combination of the meta-learning approach and image processing methods for strabismus screening. Specifically, the proposed method can quickly adapt to the strabismus screening tasks with a few training samples, which is particularly important when the number of strabismic images is limited and difficult to obtain. In addition, image processing methods extract specific features to provide supplementary information for classification, thus making up for the shortcoming that meta-learning can only extract pixel information from images. Future work will focus on exploring corner detection methods [29, 30] to locate the position of canthi more accurately and collect more strabismic images of different areas to evaluate and improve the proposed method.

## Conclusions

The present study proposed a method that combines a meta-learning approach and image processing methods for strabismus screening considering the situation of data scarcity. Experimental results showed that compared with using the meta-learning alone, the proposed method achieved much higher classification accuracy by using the supplementary information obtained by image processing methods, which demonstrated the effectiveness of the proposed method.

## Supporting information

S1 Fig Eye region images for evaluating the proposed method. (TIF)
