# Supplementary figures and images for: An improved strabismus screening method with combination of meta-learning and image processing under data scarcity
Source: PLoS One. 2022 Aug 5;17(8):e0269365. doi: 10.1371/journal.pone.0269365 (PMC9355186; doi:10.1371/journal.pone.0269365)

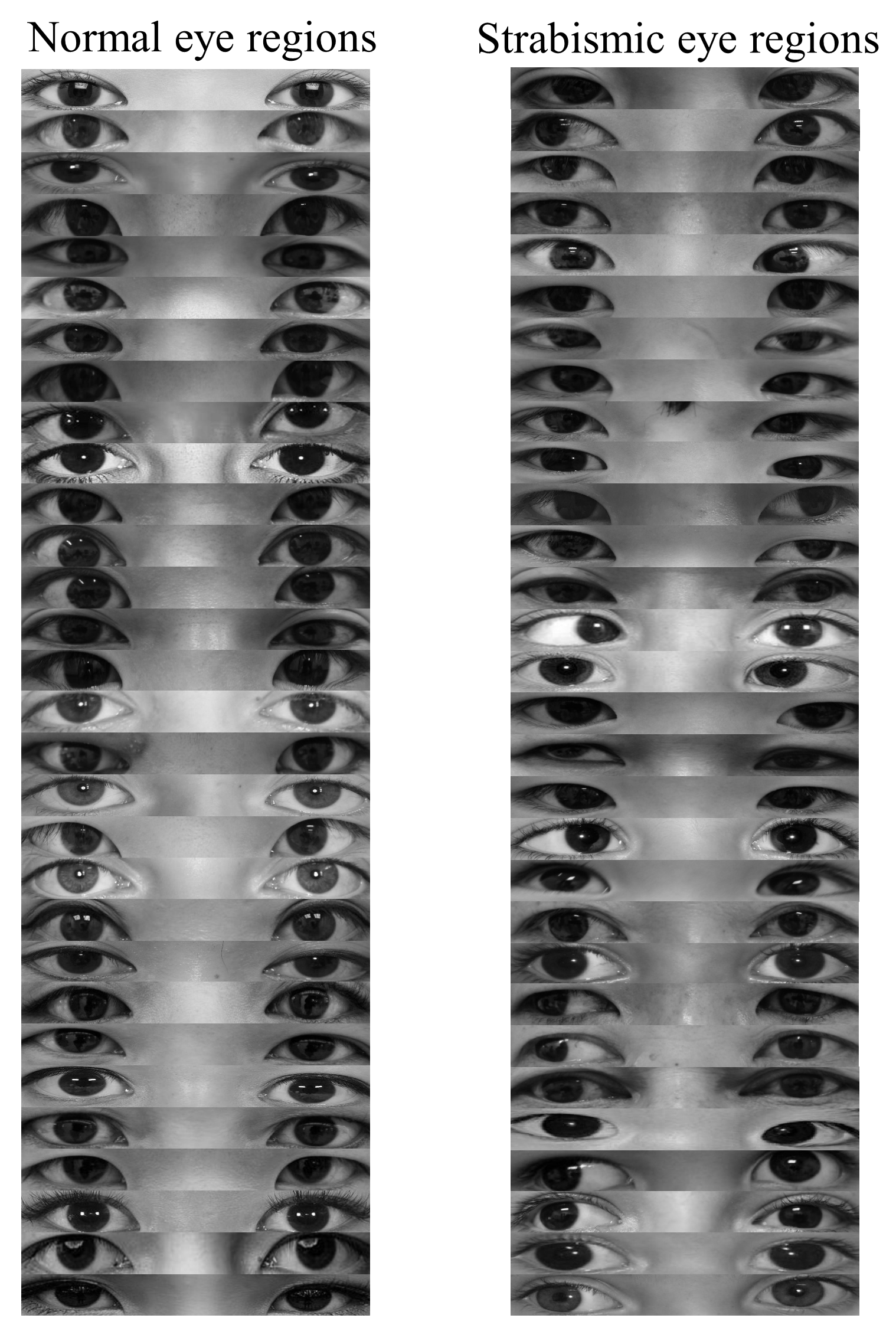

Supplement: S1 Fig — (TIF) [file pone.0269365.s001.tif]
